# A dataset of venture capitalist types in China (1978–2021): A machine-human hybrid approach

**DOI:** 10.1038/s41597-024-04108-z

**Published:** 2024-11-20

**Authors:** Jin Chen, Ruining Cao, Yifei Song, Anan Hu, Ying Ding

**Affiliations:** 1 University of Nottingham Ningbo China, Ningbo, China; 2 Fudan University, Shanghai, China

**Keywords:** Business, Economics

## Abstract

Despite escalating interest in distinguishing among various types of venture capitalists (VCs) and their roles in shaping entrepreneurship and innovation, such research remains sparse in the world’s second-largest VC market, i.e., China. To address this important gap, we have devised a machine-human hybrid approach to perform the classification task for VC types. Specifically, we have compiled a list of 49,187 VCs that made investments in China before 2021 from CVSource database, collected VC ownership information from other public sources, developed machine-learning algorithms to predict VC types, and used human coders when machine-learning failed to produce a prediction. Utilizing this hybrid approach, we have classified VCs into one of the following types: GVC (public agency-affiliated, state-owned enterprise-affiliated), CVC (corporate VC), IVC (independent VC), BVC (bank-affiliated VC), FVC (financial/non-bank-affiliated VC), UVC (university-affiliated VC), and PenVC (pension-fund-affiliated VC). We not only provide the most up-to-date database for VC types in the Chinese setting but also demonstrate how to leverage machine-learning algorithms to devise a transparent coding approach for VC-type classifications.

## Background & Summary

Scholars have increasingly emphasized the importance of differentiating among various types of venture capitalists (VCs) such as independent VCs, corporate VCs, and governmental VCs, each with unique interests in the selection, investment, and fostering of ventures. For example, unlike independent VCs (IVCs) that focus on financial returns on investments, corporate VCs (CVCs) are mainly set up to capture technology windows or harness industry growth, thus prioritizing the strategic alignment between their parent firms and their investee ventures^1^. Governmental VCs (GVCs), demonstrating remarkable growth over the past two decades, are deemed an important policy tool for bridging the equity gap and supporting high-risk start-ups that would otherwise struggle to attract private capital^2^. Additionally, VCs might also have affiliations with other entities such as banks, financial institutions, universities, or pension funds, each with divergent objectives. Due to this heterogeneity in ownership, different VCs offer distinct value-added services, resources, and network access to their investee ventures and influence the ventures towards different exit channels^3^. It is thus of great importance to develop and continually update datasets that can facilitate research in this area, enhancing our understanding of the nuanced roles of various VCs in nurturing entrepreneurship and innovation.

Despite the global proliferation of research on VC types^4^, there is an extreme dearth of such research in the context of China, the world’s second-largest VC market, partly because of the scarcity of high-quality datasets differentiating among types of VCs. For example, as Dushnitsky and Yu (2022, p.1, p.4) argued, “most of what we know about CVC investors … is based on data from the so-called developed world, and especially the United States and Western Europe,” while “the Chinese setting can offer insights into different evolutionary paths” of CVC activities^1^. While the leading databases in China (e.g., CVSource, PEdata, and ITJuzi) have provided tags to annotate a few types of VCs (e.g., GVC, CVC), the tags are either incomplete or not up to the standard of research rigor. In an effort to mitigate this data limitation, Dushnitsky and Yu^1^ have compiled a large dataset of CVCs in China by integrating multiple data sources; however, this proprietary dataset is not publicly accessible^1^. Similarly, prior studies on GVCs in China have manually collected and consolidated data regarding VCs’ government ownership, but these datasets are typically reserved for private usage^5,6^. The first and sole study to have publicly disclosed data on VC types in China’s context is Chen *et al*.^7^. They manually developed a dataset categorizing 6,553 VCs according to their affiliations and made it freely available. However, this dataset only encompasses VCs active in China’s market between 2000 and 2016, rendering it inadequate for analyzing the dramatic changes in VC activities in recent years. For instance, the influx of new VCs into China’s market continued after 2016, resulting in a population of over 49,000 VCs as of 2021. Consequently, there remains a conspicuous absence of up-to-date, high-quality datasets delineating the types of VCs that have invested in China’s market.

More importantly, previous research on China’s datasets has leaned heavily on manual coding, a laborious and time-consuming method^1,5,6^. They use public sources (e.g., WIND, China’s State Administration for Industry and Commerce, and VCs’ websites) to discern the ownership of each VC. Such a manual approach suffers from low levels of transparency and is not able to conduct data/results triangulation. As Dushnitsky and Yu (2022, p.18) commented, “We point to an important issue associated with data availability in the Chinese setting. We find that CVC patterns based on data from one database are often not replicated when testing a similar specification using a different database.” In other words, it is difficult to triangulate VC-type data from manually coded databases because of the inconsistency of their classification standards and low transparency.

Machine-learning-based technologies allow the development of a more reliable, consistent, and transparent dataset. Recent advancements in machine-learning algorithms have showcased their superior capabilities in performing classification tasks^8^. Developing a customized algorithm for VC-type classification and making it open to the public would largely help researchers increase the efficiency of data processing and the reliability of the findings. This is particularly pertinent given the exponential growth of VC numbers from thousands to tens of thousands in recent years, rendering manual coding nearly infeasible for completing the necessary tasks.

Using a hybrid approach that combines machine-learning-based and manual coding techniques, we have developed a comprehensive dataset, termed ChinaVCtype*,* that classifies VC types based on their affiliations as of 2021. Specifically, we assembled a list of VCs that had invested in China before the end of 2021, sourced from the CVSource database, a leading database on VC investment in China^9^. We then obtained their ownership details, such as shareholders, shareholder equity, and shareholder business scope, from public sources like Qichacha, a leading database on business administration information in China. In alignment with previous studies^7^, we designed a multi-step process to categorize each VC into one of the following types: GVC which includes VCs affiliated with public agencies or state-owned enterprises, CVC, independent VC (IVC), bank-affiliated VC (BVC), financial/non-bank-affiliated VC (FVC), university-affiliated VC (UVC), and pension-fund-affiliated VC (PenVC). The majority of this classification task is undertaken by machine-learning algorithm. If the algorithm can generate predictions, human coders are invited to double-check its coding quality. Whenever the algorithm is indecisive in predictions, we resort to human coders. A series of validation tests have been conducted by comparing the results of this newly developed dataset with prior studies, affirming its high quality.

This study makes three important contributions. First, the dataset we have developed can foster a wide range of topics concerning VC types and entrepreneurship in the context of China. This includes concentrating on a specific VC type (e.g., GVC, CVC), comparing different VC types (e.g., GVC vs. CVC vs. IVC), and assessing the generalizability of VC analyses from developed countries to the Chinese setting. Second and more importantly, going beyond prior studies that struggle to triangulate VC-type data from multiple Chinese VC databases due to low transparency and low consistency of those databases’ classification criteria, we have devised a machine-human hybrid approach that makes the VC-type classification process open, transparent, and consistent. By doing so, our study offers a unified VC-type data platform for future research to replicate, compare, and extend prior findings. Third, leveraging the machine-human hybrid approach, we not only provide a methodological foundation for future scholars to apply the data from various platforms on a large scale but also underscore the potential of machine-learning algorithms to assist scholars in performing extensive classification tasks.

## Methods

Following prior literature, we develop our ChinaVCtype dataset by classifying VCs to seven types, including *GVC (public agency-affiliated or SOE-affiliated VC), CVC, IVC, BVC, FVC, UVC*, and *PenVC*^4,7^. We adopted prior studies’ coding schemes whenever possible and made adaptation when necessary. We have developed machine learning algorithms to make predictions and invited human coders to conduct the classification tasks when machine learning algorithms failed to generate a prediction for a VC’s type. The whole coding process is to be elaborated in the section “Data coding scheme.”

### Data collection

To generate a list of VCs, we started with the population of VCs that have invested in China’s market as of 2021. In this study, we have adopted a broad definition of VC, referring to entities that make risky equity investments in entrepreneurial ventures. Consequently, the scope of VC in our study encompasses not only the narrowly defined VC but also private equity (PE), and even corporations that directly invest venture capital in ventures. This is because “a clear distinction between venture capital and private equity (PE) is lacking in China”^9^, and we have observed that many corporations may not yet have established their CVC entities to serve as an extension for their corporate venturing activities. Following this broad definition, in the CVSource database, there were 49,187 VCs that have made investments in China as of 2021, and the first deal appeared to be in 1978. Thus, we develop VC types for these 49,187 VCs in China’s market between 1978 and 2021.

VCs’ basic information (e.g., VC name, age, location) was collected from CVSource. Information on VCs’ first-level shareholders (e.g., shareholder name, equity ratio) was collated from Qichacha’s professional version (pro.qcc.com). For those VCs that were not captured by Qichacha, we checked other public sources (e.g., Tianyancha, Bloomberg, Crunchbase, VC official websites, press releases, media reports) to gather information. If any inconsistency across sources was detected, we followed Bertoni and Martí^10^ to triangulate between multiple sources.

### Data coding scheme

**Fig. 1:**
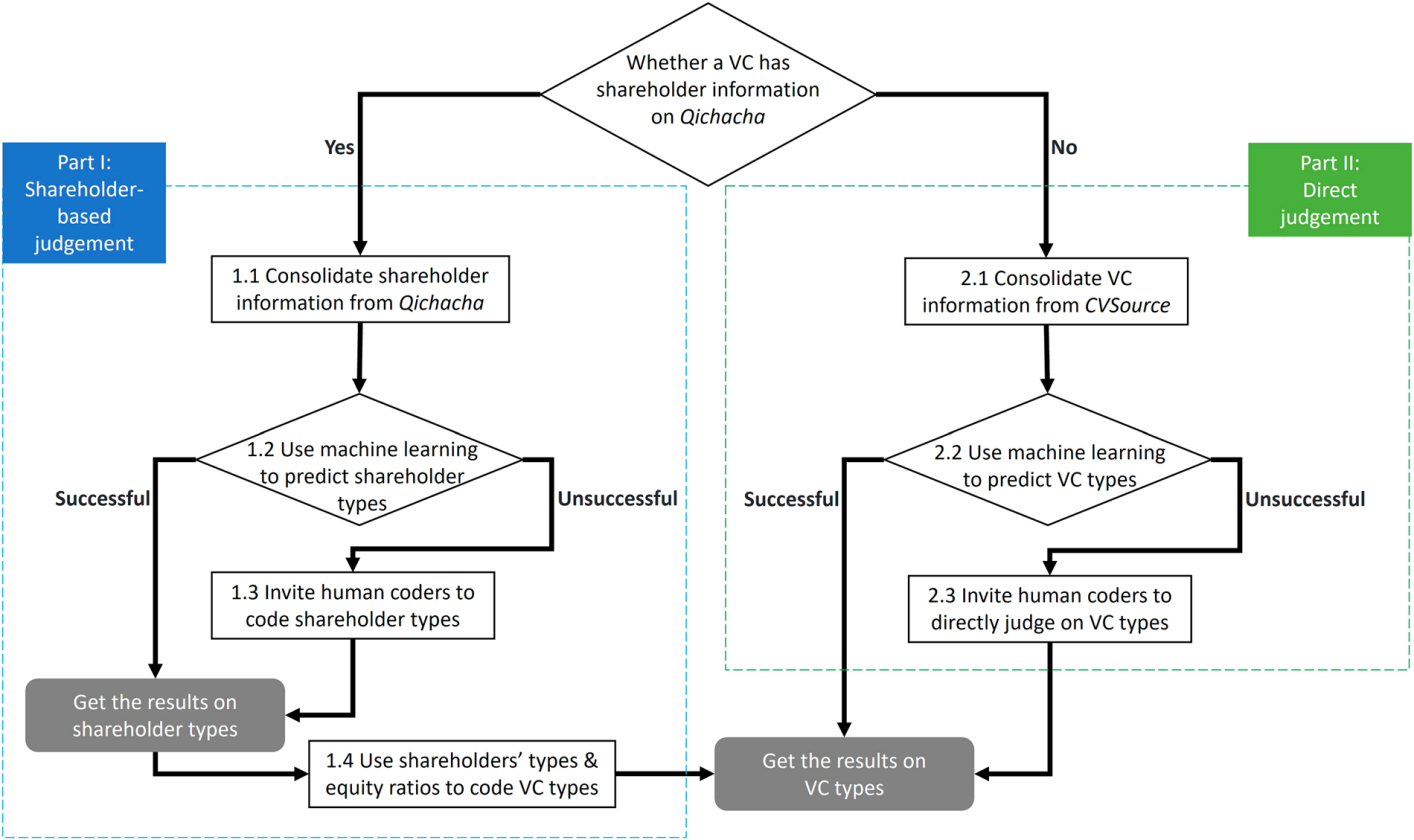
Coding process. This figure shows the coding process to determine VC types.

Figure 1 elucidates the coding process utilized to categorize various types of VCs. This process is bifurcated into two sections. Part I pertains to VCs possessing shareholder information such as first-level shareholder names and their respective equity in VCs. For these VCs, we formulated a method called “shareholder-based judgement.” This method initially employs machine learning or human coders to classify each shareholder’s type. Subsequently, the shareholder’s type and equity data are used to categorize the VC’s type. Part II addresses VCs that lack shareholder information. For these VCs, we employed a method referred to as “direct judgement.” In this approach, machine learning or human coders are used to directly determine the specific category to which a VC belongs.

#### Part I. Shareholder-based judgement

Based on the VC list from CVSource, we consolidated their full names and searched in Qichacha for their shareholder names, which resulted in 119,760 unique shareholders for 36,860 VCs. We collected the basic information of shareholders (e.g., business scope, equity ratio) and developed a four-step (Steps 1.1–1.4) approach to determine VC types, as described below.

#### Step 1.1 Consolidate shareholder information from Qichacha

We gathered basic information of VCs’ shareholders from Qichacha, including shareholder names, registered capital, ranges of the number of employees, business categories, locations, business scope, introduction, and industries. This data included numerical, ordinal, discrete, and textual data, requiring different pre-processing techniques. We need to pre-process these various types of data to be recognized by machines.

First, regarding numerical data (e.g., registered capital), we transformed them into ordinal data by using the tenth quantile as the range of classification.

Second, as for ordinal and discrete data (e.g., ranges of the number of employees, business categories, locations, industries, registered capital transformed), we used one-hot encoding to transform them to be in the dummy format^11^.

Third, considering the nature of Chinese context, we conducted multiple steps to pre-process textual data: We began with removing special symbols and unnecessary words (such as punctuation and messy code) and keeping Chinese characters, English characters, and numbers only. Next, we adopted the enterprise information dictionary and finance dictionary from Jieba (https://github.com/fxsjy/jieba), which is the most advanced word segmentation tool for Chinese, to segment words in each sentence, and applied stopwords lists from Harbin Institute of Technology to remove unimportant words or connectives^12^. After that, each shareholder’s name, business scope, and introduction were processed by TF-IDF (Term Frequency-Inverse Document Frequency), a well-known weighting technique in information retrieval and text mining that can effectively measure the similarity between sentences^13^. Following the application of TF-IDF, each sentence was transformed into a vector. However, the high dimension of each vector poses a challenge for subsequent machine learning. To address this issue, we applied the low variance filtering method, which helps delete low-variance variables that have little influence on machine learning’s prediction target. This method allows us to minimize the number of dimensions of the vectors and focus on key differences between each sentence. After data pre-processing, we obtained the representation of textual data.

#### Step 1.2 Use supervised machine learning to predict shareholder types

According to previous studies, there are eight mutually exclusive types of shareholders: “public agency, corporate, bank, financial, university, professional, person, and pension.”^7^ Machine learning is applied to differentiating among the first six types because they have plenty of textual information for algorithms to learn, while for person and pension we mainly applied human coders because it is faster to filter individual names by human coders and because China has very few pension funds.

In addition, for a shareholder categorized as corporate, bank, financial, or professional types, it may have another level of label denoting whether it is state-owned enterprise (SOE)^7^. We also used machine learning to learn this feature and make prediction on the SOE label.

#### Step 1.2.1 Sample split

The type of machine learning employed in this study is supervised machine learning, in which the algorithm first learns from a pre-labelled training dataset, predicts the labels of a test dataset, and is only applied to new data prediction if the prediction performance on the test dataset is deemed acceptable. In this sense, the training dataset is for a machine learning model to train its parameters, and the test dataset is to test the generalization ability of the model.

To train the machine learning model, a sample with correctly labelled shareholder type (e.g., public agency, corporate, bank, financial, university, professional, SOE) and feature information (e.g., shareholder names, registered capital, ranges of the number of employees, locations, business scope, introduction, and industries) is needed. For this purpose, we resorted to Chen *et al*.’s VCAC dataset^7^. They provided us the manually coded types of VCs’ shareholders between 2000 and 2016, but they did not have shareholders’ feature information. To obtain the features for prediction, we searched in Qichacha using these shareholders’ names and obtained 6,404 unique shareholders with complete information. We divided these 6,404 shareholders into a training dataset and a test dataset as 80% (5,123 shareholders) and 20% (1,281 shareholders).

#### Step 1.2.2 Model training

In total, we adopted six classical prediction models including Decision Tree, Random Forest, SVM, KNN, MLPClassifier, and Xgboost for prediction, using Python software. The metrics of prediction performance include precision, recall, and F1-score. Among the metrics, precision represents the percentage of how many positive cases predicted-to-be are with truly positive labels in the test dataset^14^. In contrast, recall reflects how many truly positive cases have been recognized by the model as positive in the test dataset^14^. F1-score synthesizes these two metrics and reflects the comprehensive performance of model prediction^15^. Thus, we used F1-score to illustrate the comparison of prediction performance. As shown in Table 1, Xgboost demonstrated superior predictive performance, with the highest accuracy consistently across all types. Thus, Xgboost model was used to predict shareholder types.

**Table 1:** F1-scores of six models predicting shareholder type based on the results of test dataset.

Type to predict	Prediction Models					
	Decision tree	Random Forest	SVM	KNN	MLPClassifier	Xgboost
1 (Public agency)	0.87	0.90	0.26	0.88	0.71	**0.97**
2 (Corporate)	0.85	**0.91**	0.62	0.67	0.72	**0.91**
3 (Bank)	0.00	0.00	0.00	0.00	0.00	**1.00**
4 (Financial)	0.48	0.40	0.03	0.18	0.00	**0.61**
5 (University)	0.91	0.67	0.40	**1.00**	0.00	**1.00**
6 (Professional)	0.82	0.87	0.64	0.73	0.63	**0.88**
7 (SOE)	0.56	0.40	0.57	0.55	0.60	**0.75**

It reports the F1-scores of six models that are used to predict shareholder types in the test dataset. *Note*: Each type’s prediction is independent of other types’; in other words, a shareholder may be labelled positive for two or more types.

The F1-score analysis of Xgboost reveals that Type 4 (Financial) records the lowest score, suggesting a low accuracy in predicting this type. A detailed manual investigation was carried out, revealing that the inadequate prediction performance is due to the resemblance in the text of Type 2 (Corporate), Type 4 (Financial), and Type 6 (Professional). As an illustration, suppose a shareholder’s business scope contains “investment and engagement in industrial projects (投资兴办实业).” Because it mentions industrial projects, the shareholder should be categorized as Type 2 (Corporate). However, the term “investment” in the business scope may lead to the misclassification of the shareholder as Type 4 (Financial) or Type 6 (Professional). Another example would be, in the introduction of Type 6 (Professional) shareholders, terms such as “venture capitalist (风险投资)” and “business consulting (管理咨询)” are frequently used, which also appear in the introduction of Type 4 (Financial) firms. To enhance the accuracy of the model’s predictions, it is imperative to implement additional rules to differentiate between Type 2 (Corporate), Type 4 (Financial), and Type 6 (Professional).

#### Step 1.2.3 Keyword-based prediction

To tackle the issues mentioned earlier, we manually screened the cases with low prediction power and implemented additional heuristics specifically designed for Type 2 (Corporate), Type 4 (Financial), and Type 6 (Professional) prediction, as shown in Table 2.

**Table 2:** Additional heuristics for shareholder type prediction.

Descriptions of heuristics
− Predict a shareholder as Type 4 (Financial) if any of the following keywords appears in **Firm Name**: securities, insurance, trust, financial services, mergers and acquisitions, guarantees (证券,保险,信托,金融服务,并购,担保).
− Predict a shareholder as Type 4 (Financial) if any of the following keywords appears in **Business Scope**: non-performing assets, resource development (不良资产,资源开发).
− Predict a shareholder as Type 4 (Financial) if any of the following keywords appears in **Industry**: life insurance, insurance agent services, insurance brokerage services, trust companies, credit services, publicly offered securities investment funds, notary services, other futures market services, reinsurance, commercial banking services, securities market management services, securities and futures regulatory services, securities brokerage transactions services, property insurance, financial company services (人寿保险,保险代理服务,保险经纪服务,信托公司,信用服务,公开募集证券投资基金, 公证服务,其他期货市场服务,再保险,商业银行服务,证券市场管理服务,证券期货监管服务,证券经纪交易服务,财产保险,财务公司服务).
− For a shareholder that is predicted as both Type 2 (Corporate) and Type 4 (Financial), we label it as Type 4 (Financial) if any of the following keywords appears in **Firm Name**: investment, assets, futures, capital, finance, financial leasing (投资,资产,期货,资本,金融,融资租赁).
− For a shareholder that is predicted as both Type 4 (Financial) and Type 6 (Professional), we label it as Type 6 (Professional) if any of the following keywords appears in **Firm Name**: venture capital, investment, consulting, asset management, incubation, innovation (创投,投资,咨询,资产管理,孵化,创新).
− For a shareholder that is predicted as both Type 2 (Corporate) and Type 6 (Professional), we label it as Type 6 (Professional) if any of the following keywords appears in **Business Scope**: business consulting (管理咨询).

It shows the heuristics developed by human coders to finetune shareholder type prediction after machine learning.

After incorporating the additional heuristics, the F1-score for Type 4 (Financial) increased to 0.65, signifying a noteworthy enhancement. However, this increase in F1-score is not sufficient, highlighting the need for additional human involvement in addressing the remaining cross-labelling issues.

#### Step 1.2.4 Screening cross-labels

The application of machine learning algorithms for shareholder type prediction resulted in multiple labels for a shareholder. A further analysis revealed 613 cross-labelled cases between categories, as shown in Table 3. For instance, 28 shareholders were predicted to be both an “Type 1 (Public agency)” and “Type 2 (Corporate),” and 340 were predicted to be both “Type 2 (Corporate)” and “Type 6 (Professional).”

**Table 3:** Cross-labels on shareholder-type classification by machine learning algorithms.

	2 (Corporate)	3 (Bank)	4 (Financial)	5 (University)	6 (Professional)	7 (SOE)
1 (Public agency)	28	0	0	0	0	3
2 (Corporate)		5	65	0	340	N.A.
3 (Bank)			19	0	0	N.A.
4 (Financial)				0	152	N.A.
5 (University)					0	1
6 (Professional)						N.A.

It reports the number of cross-labelled shareholders by machine learning till step 1.2.3. *Note*: Shareholders classified as corporate, bank, financial, or professional types can also bear the label of SOE. In other words, the cross-labels between SOE and any of these four types are not problematic and thus reported as “N.A.” in this table.

To address this issue, the first three authors conducted a re-screening and double-checking process for the cross-labelled cases. We utilized public sources of shareholder information, including shareholder names, official websites, press releases, media reports, and IPO prospectuses, to make the judgements. For instance, if a shareholder was predicted to be both “Type 1 (Public agency)” and “Type 2 (Corporate)” but had “Guidance Funds” in its name, human coders reclassified it as “Type 1 (Public agency).” To maintain the quality of the screening and checking process, three authors worked together on all 609 cases. In cases of disagreements, the fourth author was consulted to provide an independent perspective, and the team engaged in thorough discussions to achieve consensus.

#### Step 1.3 Invite human coders to code shareholder type

On several occasions, machine learning did not yield the anticipated results for shareholders. First, there were 602 shareholders for whom machine learning could not generate any predictions despite possessing information on the business scope. This is, in part, due to the brevity of their business scope descriptions. In this case, two human coders were brought in to perform the coding task, adhering to the human coding procedure outlined by Chen *et al*.^7^. The two coders independently classified the types of the 602 shareholders, and Cohen’s Kappa statistic for their results was 0.9821, indicating a very high inter-rater reliability. In instances of disagreement between the two coders, an author, serving as the third coder, was brought in to discuss and resolve the discrepancies.

Additionally, 2,681 shareholders’ information regarding business scope was completely absent. Again, we invited two human coders to independently work on the coding task, following Chen *et al*.’s procedure^7^. Cohen’s Kappa statistic was 0.9838, indicating a very high inter-rater reliability. An author acted as the third coder to intervene to reconcile any differences in the classifications provided by the two coders.

#### Step 1.3.1 Shareholder type coding

Consistent with Chen *et al*.’s^7^ “coding on shareholder type”^7^, a two-step approach for shareholder-type coding was conducted.

First, for each shareholder, we utilized its business scope information collected from Qichacha—and if this information was missing, we used information available from public sources—to categorize it into one of the eight mutually exclusive types: public agency, professional, corporate, bank, financial, university, person, and pension. The coding scheme is shown in Table 4.

**Table 4:** Coding scheme of shareholder types by human coders.

Shareholder type	Coding scheme
Public Agency	A public authority or a non-profit organization, with executives appointed by the government. This covers public authorities from central to town governments, inclusive of street offices, but excludes village committees as they are self-governing organizations in China.
Corporate	Business scope involves offering products and services unrelated to investment, financial, or banking enterprises. Examples include manufacturing, retailing, trading, importing, exporting, IT services, and business consulting.
Bank	A commercial bank, including its branches.
Financial	A financial institute, inclusive of its branches. Financial institutes here encompass entities that carry out financial activities, such as securities, insurance, trusts, hedges, non-performing asset management, and mortgages.
University	A university or college that offers higher education.
Professional	Business scope involves VC investment and/or VC-related services, such as VC investment consulting.
Person	An individual.
Pension	A pension fund.
Unknown	A shareholder is labelled as “unknown” when there is insufficient information to determine its type as per the above classifications, resulting in 192 cases in this category.

Adopted from Chen *et al*.^7^.

Second, for shareholders categorized as corporate, bank, financial, or professional types, we also assessed whether it is SOE. Specifically, we adhered to the procedure outlined by Chen *et al*.^7^ for “coding the SOE nature of shareholders.”^7^ We based our judgement of the SOE classification on two established documents they adopted, namely, “Measures for the Supervision and Administration of the Transactions of State-owned Assets of Enterprises (Order No. 32)” and “Measures for the Supervision and Administration of State-owned Equity of Listed Companies (Order No. 36).”

#### Step 1.4 Use shareholders’ type and equity ratio to code VC type

With the predicted/coded results on shareholder type and the collected information about share ratio, we proceeded with VC-level type classification. Following prior literature^7^, we created two modes of coding, namely, “non-exclusive VC type coding” and “mutually exclusive VC type coding.” This step was done by manual coding via Stata software.

Non-exclusive VC type coding is to account for research needs drawing from theories that concern the diversity of shareholders (e.g., resource-based view, agency theory, institutional logics theory). For example, a VC can tap into a variety of resources if it has multiple types of shareholders or may face different pressures from different types of shareholders, such as a public agency, corporate, and bank. Consequently, a VC can be associated with multiple affiliations that together shape its investment strategies. The coding scheme for non-exclusive VC type coding is adopted from prior literature, as shown in Table 5.

**Table 5:** Coding scheme of non-exclusive VC type for VCs with shareholder information available.

Non-exclusive VC type	Coding scheme
GVC	Has a shareholder that is labelled as public agency or SOE. We have separately included the two types as well as the information regarding the equity ratio owned by each of these two types.
CVC	Has a shareholder that is labelled as corporate.
IVC	Has a shareholder that is labelled as either professional or person.
BVC	Has a shareholder that is labelled as bank.
FVC	Has a shareholder that is labelled as financial.
UVC	Has a shareholder that is labelled as university.
PenVC	Has a shareholder that is labelled as pension.

Adopted from Chen *et al*.^7^.

Mutually exclusive VC type coding is to accommodate research needs that concern the heterogeneity at VC level, such as the diversity of VCs in a syndicate. In this case, it would be better if a VC is affiliated with a unique type, and every type is mutually exclusive. For this aim, we created a mutually exclusive coding scheme of VC type following prior literature^7^. The coding follows a sequential process, as shown in Table 6.

**Table 6:** Coding scheme of mutually exclusive VC type for VCs with shareholder information available.

Mutually exclusive VC type	Coding scheme
GVC	Meet two criteria: (1) has a shareholder that is labelled as public agency or SOE, and (2) the total equity shares owned by public agency and SOE shareholders are greater than that of any other type of shareholders (or the total number of public agency and SOE shareholders is greater than that of any other type of shareholders if the equity ratio information is not available).
CVC	Meet two criteria: (1) the VC is not classified as GVC, and (2) the VC has a unique corporate shareholder that owns over 99% of its equity or if the VC’s business scope involves offering products and services.
IVC	Either meet criteria (1) and (2) or meet criteria (1) and (3): (1) the VC is not classified as GVC or CVC, (2) professional and person shareholders own the largest total equity (or are the largest group of shareholders if the equity ratio information is not available), and (3) the VC has more than two corporate shareholders but none of them owns over 99% of its equity.
BVC	Meet two criteria: (1) the VC is not classified as GVC or CVC, and (2) bank shareholders own the largest total equity (or are the largest group of shareholders if the equity ratio information is not available).
FVC	Meet two criteria: (1) the VC is not classified as GVC or CVC, and (2) financial shareholders own the largest total equity (or are the largest group of shareholders if the equity ratio information is not available).
UVC	Meet two criteria: (1) the VC is not classified as GVC or CVC, and (2) university shareholders own the largest total equity (or are the largest group of shareholders if the equity ratio information is not available).
PenVC	Meet two criteria: (1) the VC is not classified as GVC or CVC, and (2) pension shareholders own the largest total equity (or are the largest group of shareholders if the equity ratio information is not available).

Adopted from Chen *et al*.^7^.

#### Part II. Direct judgement

After we found shareholder information for 36,860 VCs and classified their types based on shareholder information in Part I, we were left with 12,729 VCs that had no shareholder information on Qichacha. They could be individuals, VCs registered out of Mainland China, or VCs that had deregistered, thus lack of record in Qichacha. For VCs with missing shareholder information, we collected their own information from CVSource and developed a three-step (Steps 2.1–2.3) approach to determine their types, as described below.

#### Step 2.1 Consolidate VC information from CVSource

We gathered each VC’s basic information from CVSource, including VC names, business categories, investment rounds, locations, introduction, and industries. This data included ordinal, discrete, and textual data, requiring different pre-processing techniques. We pre-processed these various types of data for machine learning.

First, regarding ordinal data (e.g., investment rounds), we transformed them into discrete data by using the tenth quantile as the range of classification. Second, as for ordinal and discrete data (e.g., locations, industries, registered capital transformed), we used one-hot encoding to transform them to be dummy variables. Third, we pre-processed textual data, similar to what we did to the textual data of shareholders: We kept Chinese or English characters and numbers only. Next, we adopted two dictionaries (i.e., enterprise information dictionary, finance dictionary) from Jieba to segment words and applied stopwords to remove unimportant words or connectives. After that, we used TF-IDF to process each VC’s name and introduction, followed by low variance filtering to delete low-variance variables. After data pre-processing, we obtained the representation of textual data.

#### Step 2.2 Use supervised machine learning to predict VC types

The machine learning algorithm is capable of predicting the type of a VC only for mutually exclusive VC type but struggles to generate predictions for non-exclusive VC type. This limitation arises from the difficulty faced by the algorithm in learning and predicting the various affiliations a VC might have in the absence of shareholder information. Thus, we employed machine learning to implement a mutually exclusive coding scheme for VC types. Specifically, the machine identifies whether a VC falls under the categories of GVC, CVC, IVC, BVC, FVC, UVC, PenVC, or if it’s unable to classify a VC into any category.

#### Step 2.2.1 Sample split

We used Chen *et al*.’s VCAC dataset^7^, obtained 6,379 VCs that had basic information from CVSource as well as an assigned VC type, and divided this sample into a training dataset and a test dataset as 80% (5,103 VCs) and 20% (1,276 VCs).

#### Step 2.2.2 Model training

Similar to shareholder-level prediction, at VC level we applied Xgboost prediction model because it outperformed other models, with the highest accuracy consistently across all types. Table 7 listed the details.

**Table 7:** F1-scores of Xgboost predicting VC type based on the results of test dataset.

Type to predict	Xgboost
1 (GVC)	0.80
2 (CVC)	0.82
3 (IVC)	0.85
4 (BVC)	0.60
5 (FVC)	0.51
6 (UVC)	0.67
7 (PenVC)	N.A.

It reports the F1-scores of the Xgboost model predicting VC type in the test dataset. *Note*: Each type’s prediction is independent of other types’; in other words, a VC may be labelled positive for two or more types. The model did not predict any VC to be PenVC, thus the F1-score for PenVC is N.A.

#### Step 2.2.3 Keyword-based prediction

Similarly, for cases with low prediction power in Step 2.2.2, we implemented some heuristics specifically designed for Type 2 (CVC), Type 3 (IVC), Type 4 (BVC), and Type 5 (FVC) prediction, as shown in Table 8. By manually developing some additional heuristics as outlined in Table 8, the F1-scores exhibited notable improvements. Specifically, the F1-score increased to 0.86 for Type 2 (CVC), to 0.88 for Type 4 (BVC), and to 0.55 for Type 5 (FVC), indicating a noteworthy enhancement. We did not develop any heuristics for Type 6 (UVC) because its low F1-score was mainly due to its limited number of observations in the training dataset instead of its overlapping with other categories, and human screening was applied to improve its categorization.

**Table 8:** Additional heuristics for VC type prediction.

Descriptions of heuristics
− Predict a VC as FVC if any of the following keywords appears in **Firm Name**: securities, insurance, trust, financial services, mergers and acquisitions, guarantees (证券,保险,信托,金融服务,并购,担保).
− Predict a VC as BVC if any of the following keywords appears in **Firm Name**: bank (银行).
− For a VC that is predicted as both CVC and FVC, we label it as FVC if any of the following keywords appears in **Firm Name**: investment, assets, futures, capital, finance, financial leasing (投资,资产,期货,资本,金融,融资租赁).
− For a VC that is predicted as both CVC and IVC, we label it as IVC if any of the following keywords appears in **Firm Name**: venture capital, investment, consulting, asset management, incubation, innovation (创投,投资,咨询,资产管理,孵化,创新).

It lists the heuristics developed by human coders to finetune VC type prediction after machine learning.

#### Step 2.2.4 Screening cross-labels

The machine learning algorithm may suggest multiple types for a VC. Upon further examination, there were 5 instances of cross-labelling between VC categories, as shown in Table 9. To address the issue of cross-labelled VCs, we followed the same double-checking process as we did for shareholders in Step 1.2.4, using public sources of VC information such as VC names, official websites, press releases, and media reports.

**Table 9:** Cross-labels on VC-type classification by machine learning algorithms.

	2 (CVC)	3 (IVC)	4 (BVC)	5 (FVC)	6 (UVC)	7 (PenVC)
1 (GVC)	0	0	0	0	0	N.A.
2 (CVC)		1	0	2	0	N.A.
3 (IVC)			0	2	0	N.A.
4 (BVC)				0	0	N.A.
5 (FVC)					0	N.A.
6 (UVC)						N.A.

It reports the number of cross-labelled VCs by machine learning till step 2.2.3.

#### Step 2.3 Invite human coders to directly judge on VC type

So far, out of the 12,729 VCs lacking shareholder information, 5,506 VCs were successfully categorized by machine learning algorithms. For the rest (7,223 VCs) that did not have adequate VC level information or machine learning could not yield the anticipated results for their types, two independent human coders were invited to directly determine VC types, following a two-step approach. Cohen’s Kappa statistic between their results was 0.9936, indicating a high inter-rater reliability. An author stepped in as the third coder to settle the disagreements between the two coders.

First, based on all the information from public sources, each human coder determined whether a VC belongs to a type, as outlined in Table 10. For example, when determining GVC, we mainly relied on Qichacha’s label for public agency or SOE and we double-checked the quality of Qichacha’s labelling. If a VC investor is labelled as public agency or SOE according to Qichacha, we directly determined it as GVC. If Qichacha does not have the information, we resorted to a set of public sources (e.g., Tianyancha, Baidu, Bloomberg, Crunchbase, Google, government websites, PEdata, ITJuzi, news reports, VCs’ official websites, Sogou) to classify whether it is GVC, adopting Chen *et al*.’s^7^ definition of GVC^7^. When determining other types of VCs, we mainly relied on information from the same set of public sources to pinpoint the VCs’ main business scope to make direct judgement on VC types.

**Table 10:** Human coders’ coding scheme of mutually exclusive VC type for VCs with missing shareholder information.

Mutually exclusive VC type	Coding scheme
GVC	An investor labelled as public agency or SOE by Qichacha or other public sources.
CVC	Business scope involves offering products and services unrelated to investment, financial, or banking enterprises. Examples include manufacturing, retailing, trading, importing, exporting, IT services, and business consulting.
IVC	An investor as an individual or whose business scope involves VC investment and/or VC-related services, such as VC investment consulting
BVC	An investor affiliated with a commercial bank, including its branches.
FVC	An investor affiliated with a financial institute, inclusive of its branches. Financial institutes here encompass entities that carry out financial activities, such as securities, insurance, trusts, hedges, non-performing asset management, and mortgages.
UVC	An investor affiliated with a university or college that offers higher education.
PenVC	An investor managing pension fund.

Adapted from Chen *et al*.^7^.

## Data Records

The ChinaVCtype dataset can be accessed through figshare.com^16^. It consists of two plain text files in CSV format, namely, “ChinaVCtype.csv” and “Alias.csv.”

The “ChinaVCtype.csv” file provides the type of 49,187 VCs that have made investments in China between 1978 to 2021. The definitions of variables are listed below:

1. VC_fullname: A string variable that denotes the full name of a VC.
2. Unified_social_credit_ID: A string variable that denotes the unified social credit ID of a VC, if available.
3. GVCagency_ne: A dummy variable that equals 1 if a VC is labelled as public agency-affiliated based on the non-exclusive VC type coding, and 0 otherwise. A missing value indicates that the information on this variable is lacking.
4. GVCagency_equity: A continuous variable that represents the total equity owned by public agency shareholders in a VC. Noteworthy, the value of GVCagency_equity could be underestimated if the equity ratio of a public-agency shareholder for a VC is missing. And a missing value of GVCagency_equity indicates that equity ratio data for the VC is not available.
5. GVCsoe_ne: A dummy variable that equals 1 if a VC is labelled as SOE-affiliated based on the non-exclusive VC type coding, and 0 otherwise. A missing value indicates that the information on this variable is lacking.
6. GVCsoe_equity: A continuous variable that represents the total equity owned by SOE shareholders in a VC. Noteworthy, the value of GVCsoe_equity could be underestimated if the equity ratio of an SOE shareholder for a VC is missing. And a missing value of GVCsoe_equity indicates that equity ratio data for the VC is not available.
7. IVC_ne: A dummy variable that equals 1 if a VC is labelled as IVC based on the non-exclusive VC type coding, and 0 otherwise. A missing value indicates that the information on this variable is lacking.
8. CVC_ne: A dummy variable that equals 1 if a VC is labelled as CVC based on the non-exclusive VC type coding, and 0 otherwise. A missing value indicates that the information on this variable is lacking.
9. BVC_ne: A dummy variable that equals 1 if a VC is labelled as BVC based on the non-exclusive VC type coding, and 0 otherwise. A missing value indicates that the information on this variable is lacking.
10. FVC_ne: A dummy variable that equals 1 if a VC is labelled as FVC based on the non-exclusive VC type coding, and 0 otherwise. A missing value indicates that the information on this variable is lacking.
11. UVC_ne: A dummy variable that equals 1 if a VC is labelled as UVC based on the non-exclusive VC type coding, and 0 otherwise. A missing value indicates that the information on this variable is lacking.
12. PenVC_ne: A dummy variable that equals 1 if a VC is labelled as PenVC based on the non-exclusive VC type coding, and 0 otherwise. A missing value indicates that the information on this variable is lacking.
13. GVC_excl: A dummy variable that equals 1 if a VC is labelled as GVC based on the mutually-exclusive VC type coding, and 0 otherwise.
14. GVC_equity: A continuous variable that represents the total equity owned by public agency-affiliated and SOE-affiliated shareholders in a VC. Noteworthy, the value of GVC_equity could be underestimated if the equity ratio of a shareholder for a VC is missing. And a missing value of GVC_equity means that equity ratio data for the VC is not available.
15. CVC_excl: A dummy variable that equals 1 if a VC is labelled as CVC based on the mutually-exclusive VC type coding, and 0 otherwise.
16. IVC_excl: A dummy variable that equals 1 if a VC is labelled as IVC based on the mutually-exclusive VC type coding, and 0 otherwise.
17. BVC_excl: A dummy variable that equals 1 if a VC is labelled as BVC based on the mutually-exclusive VC type coding, and 0 otherwise.
18. FVC_excl: A dummy variable that equals 1 if a VC is labelled as FVC based on the mutually-exclusive VC type coding, and 0 otherwise.
19. UVC_excl: A dummy variable that equals 1 if a VC is labelled as UVC based on the mutually-exclusive VC type coding, and 0 otherwise.
20. PenVC_excl: A dummy variable that equals 1 if a VC is labelled as PenVC based on the mutually-exclusive VC type coding, and 0 otherwise.
21. Unknown: A dummy variable that equals 1 if a VC is labelled as unknown VC, and 0 otherwise. For this variable, both non-exclusive and mutually-exclusive VC type coding generated the same.
22. Direct_judge: A string variable that indicates whether a VC’s type is determined at the VC level or the shareholder level. It has three possible values:
  1. “No”: This value indicates that the case is judged at the shareholder level because shareholder information is available. In other words, the coding of the case follows the procedure of Part I “Shareholder-based judgement.”
  2. “Machine Learning”: This value indicates that the case is judged at the VC level because no shareholder information is available on Qichacha, and the judgement is made by machine learning. In other words, the coding of the case follows the procedure of Part II “Direct judgement.”
  3. “Human Coding”: This value indicates that the case is judged at the VC level because no shareholder information is available on Qichacha, and the judgement is made by human coders. In other words, the coding of the case also follows the procedure of Part II “Direct judgement.” Noteworthy, when “Direct_judge” takes the value of “Human coding”, two variables—GVCagency_ne and GVCsoe_ne—have values because human coders could search for GVC’s shareholder information from other public sources.

23. Corporate_parent: A string variable that denotes a CVC’s corporate parent, if available.
24. Parent_unified_social_credit_ID: A string variable that denotes the unified social credit ID of a CVC’s corporate parent, if available.
25. Parent_industry_qcc: A string variable that denotes the industry of a CVC’s corporate parent, according to Qichacha’s industry classification.
26. Parent_listed: A string variable that indicates whether a CVC’s corporate parent is publicly listed or not. It has three possible values:
  1. “Listed”: This value indicates that the parent firm is a publicly listed firm as of July 2024.
  2. “Delisted”: This value indicates that the parent firm had been listed previously but was already delisted by the time of observation in July 2024.
  3. Blank: The parent firm is not listed yet.

27. Parent_listedmarket: A string variable that denotes the listed stock market of a CVC’s corporate parent.
28. Parent_stockcode: A string variable that denotes the stock code of a CVC’s corporate parent.

The “Alias.csv” file lists all the alias names for VCs, to further improve the matching by users. It also includes the alias names of 529 VCs that appeared in Chen *et al.’s*^7^ VCAC dataset but not in our ChinaVCtype.csv, to enhance the compatibility of our dataset. In addition, if a VC’s name is shown in ChinaVCtype.csv but not in the Alias.csv, it indicates that the VC does not have an alias in our dataset. The total number of records in Alias.csv is 43,428, with variable definitions listed below:

1. VC_fullname: A string variable that denotes the full name of a VC. This is the same name used in “ChinaVCtype.csv.”
2. Alias: A string variable that denotes the alias name of a VC. A VC could have multiple alias names, each being listed in separate rows.

## Technical Validation

To ensure the quality of the dataset, the following measures were implemented:

### Upon dataset composition: Matching VC names between multiple sources

One difficulty in developing our dataset is to match VC names among multiple sources (e.g., CVSource, Qichacha) when they adopt different forms of VC names. To start, we referred to CVSource’s deal database and compiled an initial list of 49,589 VCs that have invested in China before the end of 2021; however, most VC names are abbreviated in CVSource’s deal database. This presented a challenge in matching these names with their full versions and searching for their shareholder information in public sources.

To address this challenge and ensure the accuracy of our VC full names, we employed a multi-step approach. First, because an organization’s unified social credit ID (e.g., 91310000691612937 A, GP Capital Co., Ltd.) or its industrial and commercial registration number (e.g., 310000000096327, GP Capital Co., Ltd.) could be its unique identifier in China, we first applied these two pieces of information, if available in CVSource, to get VCs’ full names. Specifically, we collected VCs’ unified social credit ID (e.g., 91310000691612937 A, Shanghai GP Capital) and full names from CVSource’s GP (general partner) database, resulting in 13,767 VCs with such information. Second, in cases where CVSource’s GP database did not contain this information, we used VCs’ abbreviated names to search in Qichacha database. Specifically, we resorted to Qichacha’s “firm name cleaning” function as a solution. This feature, available in Qichacha’s professional version (pro.qcc.com), retrieves a firm’s full name when an abbreviated name is submitted. We submitted all variations of VCs’ names recorded in CVSource (i.e., a VC’s abbreviated name, its corresponding GP name, its corresponding LP name when it acts as a limited partner) to Qichacha’s “firm name cleaning” function. A key advantage of Qichacha’s “firm name cleaning” function is that it can provide a firm’s full current name when an abbreviated or previous name is submitted. This step has led to the successful matching of 31,106 VC full names. We also collected their unified social credit ID or industrial and commercial registration number if available in Qichacha’s “firm name cleaning.” In total, the above two steps resulted in 44,873 full names of VCs, and there were 4,716 VCs that could not be matched with CVSource’s GP or Qichacha databases using their names.

Next, we conducted a comprehensive search in Qichacha database for the shareholder information of the 44,873 VCs using their full names. Our search revealed that out of these VCs, 36,860 had available shareholder information that covered 119,760 unique shareholders, and 8,013 VCs lacked shareholder information due to their status as either foreign or deregistered entities. For the 36,860 VCs with shareholder data, we categorized them following the shareholder-based judgement procedures outlined in Part I of Fig. 1. For the 8,013 VCs devoid of shareholder information, we directly determined their types following the direct judgement procedures given in Part II of Fig. 1.

Additionally, for the remaining 4,716 VCs that could not be matched with CVSource’s GP or Qichacha databases using their names, we manually searched for their full names in public sources and directly classified them, again adhering to the procedures detailed in Part II of Fig. 1. In total, we classified 12,729 VCs directly without reliance on shareholder information.

Upon acquiring the full names of VCs, we thoroughly consolidated the list. This process uncovered that a VC might have several different names within the CVSource dataset. For instance, “杭州阿里巴巴创业投资管理有限公司” (Hangzhou Alibaba Venture Capital Management Co., Ltd.) and “阿里巴巴创投” (Alibaba VC) denote the same VC firm. After eliminating these duplicate instances, we compiled a definitive list of 49,187 unique VCs identified by their full names.

**Fig. 2:**
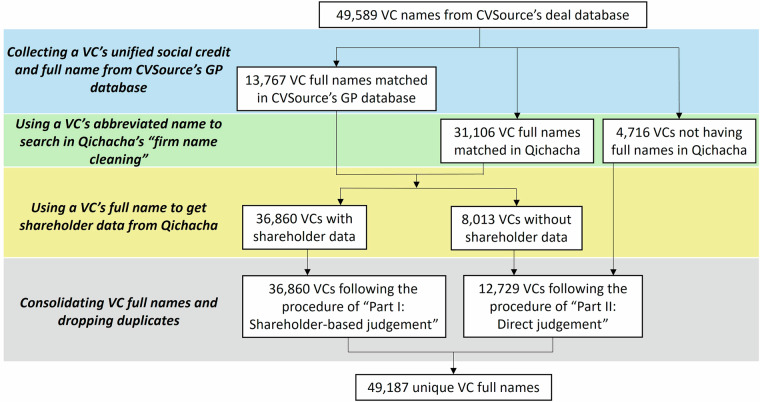
The process of identifying VC full names. This figure provides a visual representation of the entire process involved in identifying the full names of VCs.

Figure 2 depicts the entire process of identifying the full names of VCs.

### Upon direct judgement on VC types by human coders: Triangulating across coders

In directly judging the types of 12,729 VCs, 5,506 were successfully categorized by machine learning algorithms, while the remaining 7,223 that did not have complete information necessary for machine learning required further manual classification by human coders. The human coders conducted a thorough review of public sources to ensure accuracy. To maintain quality control, we formed a four-person team consisting of two master students with relevant industry experience and two authors. The third author provided training and quality checks for student research assistants until they met our standards. The research assistants then classified the VCs, with the third author conducting a double-check of any inconsistent results. If disagreements persisted, the first author was consulted to resolve the issue and achieve a final decision. This rigorous process helps ensure the quality of human coding.

### Upon the results of VC type classification: Comparing with prior studies

To verify the quality of the **ChinaVCtype** dataset, we merged it with CVSource, profiled the distributions according to VC types (e.g., numbers of VCs by type, investment deals by type), and compared the results with those in prior studies.

**Fig. 3:**
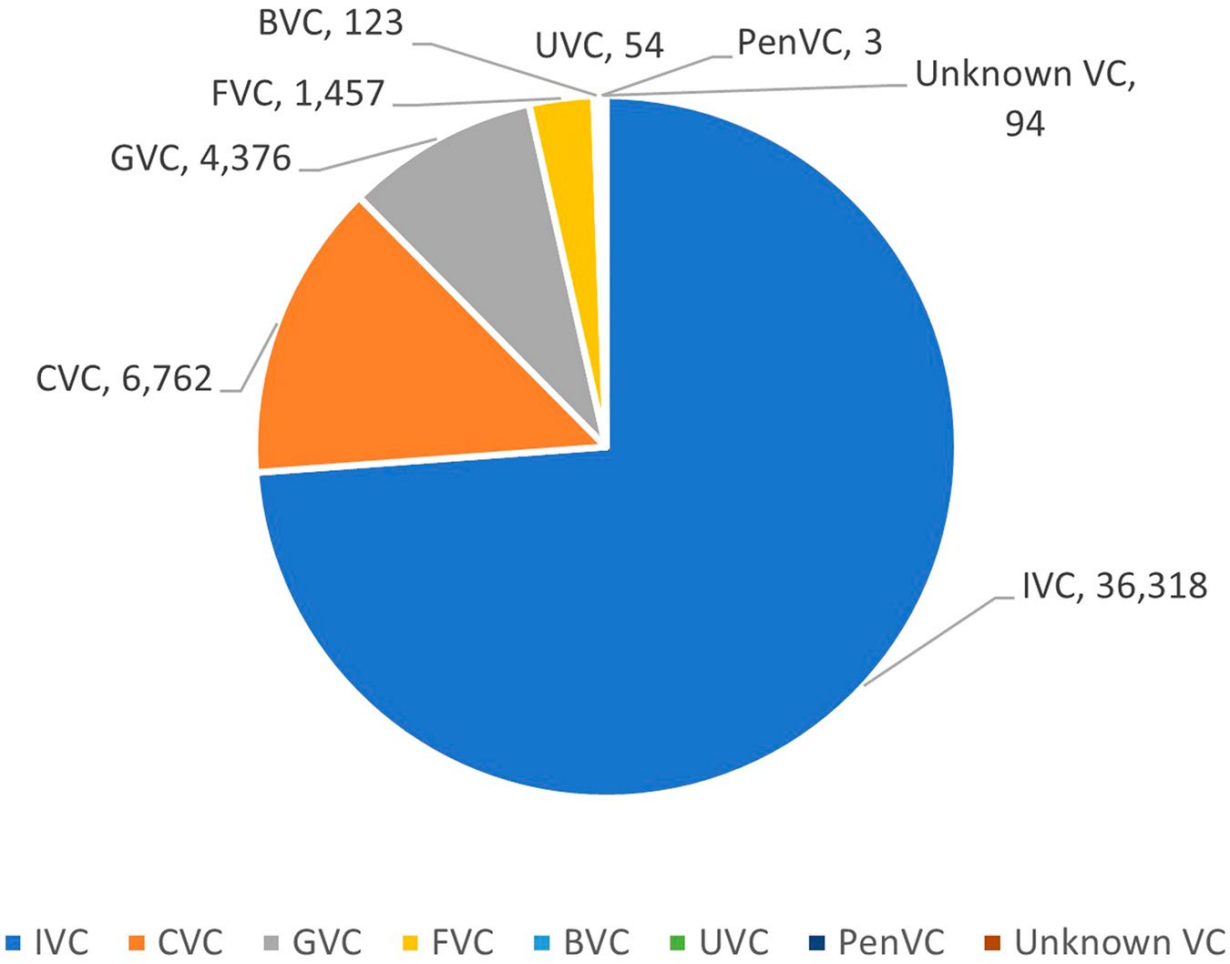
Number of VCs by affiliation. This figure reports the distribution of the number of VCs by their type in China’s market between 1978 and 2021.

Figure 3 shows the distribution of VCs by their type in China’s market between 1978 and 2021, using a mutually-exclusive coding of VC type. Out of the 49,187 VCs, 73.84% are IVCs, making them the largest group in China, followed by CVCs (13.75%), GVCs (8.90%), FVCs (2.96%), BVCs (0.25%), and UVCs (0.11%). Because only 3 PenVCs were identified, this category was omitted in the remaining analysis.

**Fig. 4:**
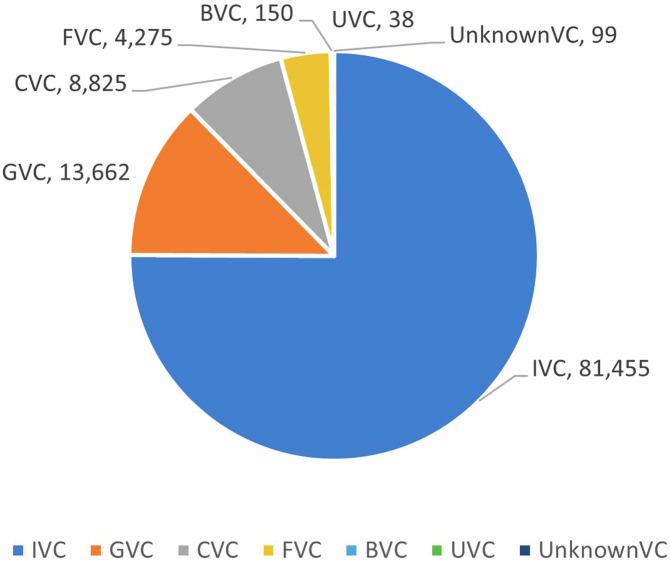
Number of VC investments by type. This figure reports the distribution of investment deals by the type of lead VC in China’s market between 1978 and 2021.

Figure 4 displays the distribution of investment deals by their lead VC’s type in China between 1978 and 2021, using a mutually-exclusive coding of VC type. As shown in Fig. 4, during this period, IVCs made the most investments (75.07%) in China’s market, followed by GVCs (12.59%), CVCs (8.13%), and FVCs (3.94%), while BVCs and UVCs contributed less than 1% each. Noteworthy, while the total number of GVCs is fewer than that of CVCs (see Fig. 3), they were more active in China’s market compared to CVCs (see Fig. 4).

**Fig. 5:**
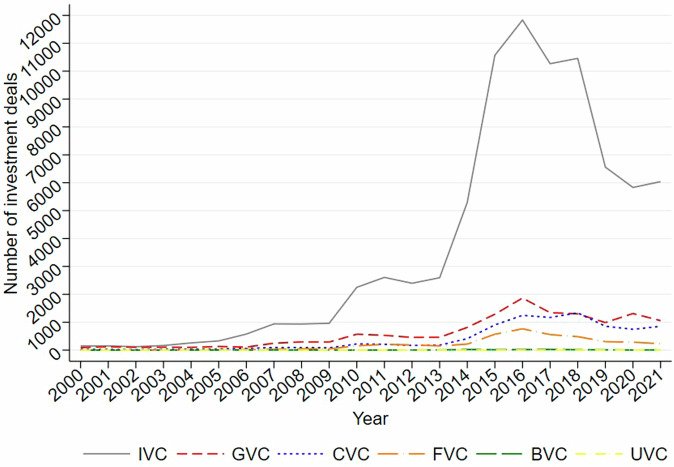
Number of investment deals by VC type. This figure reports the number of investment deals made by IVC, GVCs, CVCs, FVCs, BVCs, and UVCs in China’s market between 2000 and 2021.

**Fig. 6:**
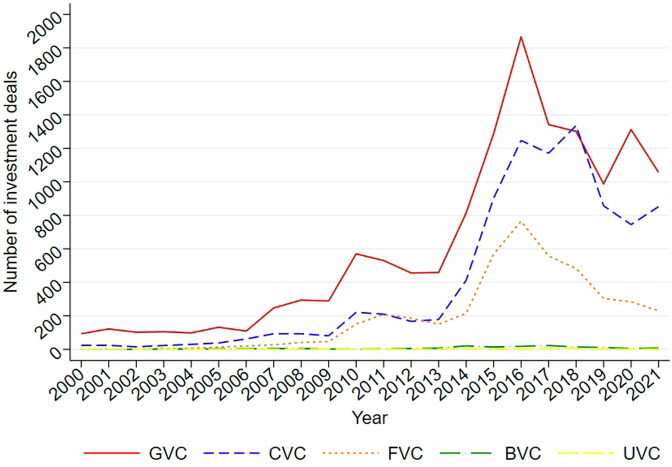
Number of investment deals by VC type (excluding IVCs). This figure reports the number of investment deals made by GVCs, CVCs, FVCs, BVCs, and UVCs in China’s market between 2000 and 2021.

Figure 5 displays the annual trend of investment deals made by each type of VC (i.e., the lead VC) in China’s market between 2000 and 2021, using a mutually-exclusive coding of VC type. Data before 2000 were dropped in the trend analysis due to their scantiness. As shown in Fig. 5, the number of deals invested by each type of VC experienced ups and downs during the reported period and crested in 2016. However, since IVC’s predominant proportion may obscure others’ trends, an extra comparison excluding IVC is shown in Fig. 6. It is now clear that both GVCs and FVCs peaked their deal numbers in 2016, while CVCs reached their peak two years later.

Next, we compared these results with previous studies that have conducted thorough manual checks on VC type to overcome the limitations of existing databases. Specifically, we focus on the results of GVC or CVC studies. While IVC is the largest group, GVC and CVC have their unique characteristics, and analyzing these two groups can better validate whether our data can well distinguish among VC types.

Regarding GVC studies in the context of China, three exemplars have been identified. Suchard *et al*. sampled 4,700 entrepreneurial firms that received first-round investments from 1,254 VCs between 1988 and 2011, and hand-collected “data on government ownership of a VC through VC profiles, web searches, and the Chinese company registration database (NECIPS).”^5^ In their sample, 240 VCs were classified as GVCs, accounting for 19.14%. For our study, we restricted our sample to all first-round investments before the end of 2011, identifying a total of 1,885 VCs, among which, 361 were GVCs, a percentage of 19.15% that is comparable to Suchard *et al*.’s observation. Additionally, Suchard *et al*. reported that “We obtain government percentage ownership data from company registration records for 78% of these [GVCs]. The average government ownership of a VC is 74%.”^5^ In our sample, 84.49% of the 361 GVCs have government percentage ownership data, and the average government ownership of a VC is 86.01%. Based on these comparisons, we find that our sample is more comprehensive than Suchard *et al*.’s (in terms of sample size and ownership data) and our descriptive statistics are consistent with theirs, reassuring the quality of ChinaVCtype dataset.

Zhang and Mayes collected a list of VCs from PEdata database between 1991 and 2010^6^. They restricted GVCs to be those owned and controlled by government agencies (i.e., excluding GVCs that were mainly owned by SOEs), and to identify their targeted GVCs, they “check[ed] the detailed description of venture capital firms in PEdata and augment[ed] the data by cross-checking the official website of each VC firm to ensure a reliable identification.”^6^ In addition, for their research purpose, they only kept ventures receiving first-round investments from a GVC or non-GVC solo investor (i.e., no syndicate), and dropped deals if investment amount information is missing. In total, they ended with “2,554 VC-backed portfolio companies, of which 436 are backed by GVCs”, translating to a GVC investment ratio of 17.07%0^6^. To mimic their sampling strategy, we restricted our sample to A-round deals that were made before the end of 2010, had only one VC investor, and had investment amount information. Also, following their definition of GVC, we kept only GVCs that were owned and controlled by government agencies by dropping GVCs with a ratio of SOE equity no less than 50%. This resulted in a sample consisting of 2,663 VC-backed ventures, out of which 463 are backed by GVCs, translating to a GVC investment ratio of 17.39%. Our results, regardless of using the total number of GVC investments or GVC investment ratio, are similar to those of Zhang and Mayes. This is remarkable, especially considering that our dataset and theirs adopted different sources (CVSource vs. PEdata) and coding methods (machine learning vs. manual coding).

In addition, Zhang^17^ collected a sample of first-round VC investments in China between 1995 and 2011 from PEdata, and manually coded if a VC was GVC or not. She defined GVCs as those established, mainly funded, and managed by the government. In her sample, out of 5,222 first-round investment deals, 1,052 were made by GVCs, resulting in a GVC investment ratio of 20.14%. Based on our dataset and using the same sampling strategy, we ended with 5,578 first-round investment deals between 1995 and 2011, of which 1,238 were invested by GVCs (with state equity ratio no less than 20%), indicating a GVC investment ratio of 22.19%. The GVC investment ratio is nearly the same across her and our samples. Moreover, in terms of syndication or not, Zhang divided GVC deals into 611 GVC-solo deals (58.10% of all GVC deals) and 441 GVC-syndication deals. In comparison, our sample contained 750 GVC-solo deals (60.58% of all GVC deals) and 384 GVC-syndication deals. The distributions of GVC sub-type investments between our sample and Zhang’s are roughly consistent, and the difference between the two might be attributed to the different sources (CVSource vs. PEdata) used to identify if a deal was solo or syndication.

Second, regarding CVC studies in the context of China, Dushnitsky and Yu’s^1^ study is the only one that has reported statistics of CVC activity in China using manually coded data^1^, which can be a benchmark for comparison. They collected data from five different sources, including ThomsonOne, CapitalIQ, CVSource, PEdata, and ITJuzi, and integrated them to form a proprietary dataset. They defined an investor as a CVC “if it was an industrial company or the affiliated sub-unit (such as a wholly-owned subsidiary or a majority-owned subsidiary) of an industrial company.”^1^ Our definition of CVC is identical to theirs, which not only includes narrowly defined strategic VC/PE investors but also covers corporates that have made CVC investments but not yet established a dedicated VC for this corporate venturing purpose. To build their dataset, Dushnitsky and Yu started with all VCs that were publicly listed (excluding real-estate and financial sectors), manually identified CVCs by using their main business scope and shareholder information, and compared their classification “with the investor’s type field in the databases and ran another check to confirm whether any CVC investors were erroneously omitted.”^1^ In addition, they dropped CVCs that made less than three deals during the observation period. As revealed by their final dataset, 173 publicly listed CVCs have disbursed 3,571 CVC investments between 2013 and 2017; on average, each publicly listed CVC made 4 deals in a year. Following their sampling strategy (i.e., excluding real-estate and financial sectors and dropping CVCs with less than three deals), our results show that 513 CVCs have made 4,129 CVC investments between 2013 and 2017; on average, each CVC made 1.6 deals in a year. That is, we discovered 340 more CVCs than Dushnitsky and Yu did (which discrepancy could be attributed to private CVCs), and our number of CVC deals was 558 more than theirs (which discrepancy may also be due to private CVC deals). This difference in the average CVC’s annual deal number is reasonable because private CVCs, on average, invested in fewer deals than publicly listed CVCs did.

Table 11 provides a summary of the comparisons made between our dataset on GVC or CVC investments and prior studies that used manual coding on VC types. We used the same definitions of GVC or CVC and followed their sampling strategies in each comparison. The results consistently show that the **ChinaVCtype** dataset is of high quality.

**Table 11:** Comparison with prior literature on GVC investments or CVC investments.

	Suchard *et al*.’s sample, 1988–2011	Our sample, 1988–2011	Zhang & Mayes’s sample, 1991–2010	Our sample, 1991–2010	Zhang’s sample, 1995–2011	Our sample, 1995–2011	Dushnitsky & Yu’s sample, 2013–2017, publicly listed CVCs	Our sample, 2013–2017, publicly listed & private CVCs
No. of VCs	1,254	1,885						
No. of GVCs	240	361						
GVC ratio	19.14%	19.15%						
No. of VC deals			2,554	2,663	5,222	5,578		
No. of GVC deals			436	463	1,052	1,238		
GVC deal ratio			17.07%	17.39%	20.14%	22.19%		
GVC-solo ratio					58.10%	60.58%		
No. of CVC deals							3,571	4,129

The columns report the distributions based on previous studies and the corresponding distributions based on our dataset. *Note*: Different studies may adopt different definitions of GVC. We followed their definitions when making the comparisons.

## Usage Notes

The CSV files are encoded in UTF-8 and separated by commas. The VC names are in Chinese.

We will maintain the dataset in the future and may add new features or variables as they emerge from our research or are suggested by the users of our dataset. In this case, a log file will be provided to inform users of any new features added to the dataset.

### Implications

Building on Chen *et al*.’s previously developed VCAC dataset^7^ and leveraging more advanced machine learning techniques, in this study, we have developed a new dataset, i.e., ChinaVCtype, to provide the classification for VC types in China’s market. The ChinaVCtype dataset has advantages over the VCAC dataset in two important ways.

First, ChinaVCtype covers a wider range of VCs in China’s market than VCAC because we have adopted a broader definition of VC to cover not only narrowly defined VCs/PEs but also other organizations that directly invest venture capital in ventures. In contrast, Chen *et al*.^7^ followed a narrow definition of VC that mainly covered VCs/PEs in CVSource. Adopting a broader definition of VC is extremely valuable because there is a significant increase in organizations that have made VC investments in China but have not yet established their VC arms dedicated to this purpose. This echoes Dushnitsky and Yu’s (2022, p.10) recent observation on China’s CVC activities, in which study they also included “an industrial company or the affiliated sub-unit (such as a wholly-owned subsidiary or a majority-owned subsidiary) of an industrial company” as a CVC even if the company has not established a professional arm to manage its VC investments^1^. To illustrate the potential of adopting a broader definition of VCs that follows ongoing research trends, Table 12 shows a comparison between VCAC and ChinaVCtype in terms of applying different VC definitions. If we adopt the same definition as Chen *et al*.^7^ by using CVSource’s label on VCs/PEs, the number of VCs between 2000 and 2016 is 10,932, slightly more than Chen *et al*.^7^; the discrepancy may be attributed to CVSource’s continuous updates on their VC/PE labels. It shows the wider coverage of ChinaVCtype compared to VCAC. Moreover, if we adopt a broader definition, this number will increase to 23,726. The comparison becomes even more significant between 2017 and 2021: 13,622 narrowly defined VCs versus 33,906 broadly defined VCs, partially due to China’s continuous policy support towards entrepreneurship and the launch of the STAR Market (Science and Technology Innovation Board) in 2019. Given the dramatic increase in organizations that play the role of VCs in providing venture capital to startups, investigations into these broadly defined VCs can provide a more complete understanding of China’s VC market and its interplay with entrepreneurship and innovation.

**Table 12:** Comparison between VCAC and ChinaVCtype.

Year	No. of narrowly defined VCs that invested during the specified years, in VCAC	No. of narrowly defined VCs that invested during the specified years, in ChinaVCtype	No. of broadly defined VCs that invested during the specified years, in ChinaVCtype
1978–1999	N.A.	120	223
2000–2016	6,379	10,932	23,726
2017–2021	N.A.	13,622	33,906
*Total:* 1978–2021		18,871	49,187

The columns report the number of VCs in VCAC that followed a narrow definition and the number of VCs in ChinaVCtype that adopted a narrow or broader definition of VCs.

Second, ChinaVCtype includes a wider array of variables compared to VCAC to enhance dataset quality. For example, VCAC utilizes the full names of VCs as the index for users’ matching, which may, however, change over time. To address this issue, ChinaVCtype not only provides the full names of VCs but also includes their unified social credit ID if such information is accessible on Qichacha, in order to assist users in aligning their data with our VC classification. Another example is the corporate parents of CVCs. While VCAC lists the names of corporate parents for CVCs, it only includes 368 corporate parents. In contrast, ChinaVCtype expands this number to 3,508—approximately ten times that of VCAC. Beyond just names, ChinaVCtype provides additional details about corporate parents, such as their unified social credit ID, industry sector, public listing status, and the markets where they are listed. The increased sample of corporate parents and these additional variables pertaining to CVCs can foster new avenues of research in this field.

Despite its notable advantages over VCAC, ChinaVCtype does have some limitations of which researchers should be aware. Unlike VCAC, which is a panel dataset accounting for changes in a VC’s type due to shareholder changes over time, ChinaVCtype remains cross-sectional for some reasons. We acknowledge that it is meaningful to reveal type dynamics by using a panel approach. Take 浙江浙大友创投资管理有限公司 (Zhejiang Zheda Youchuang Investment Management Co., Ltd) from the VCAC dataset as an example. It started as a UVC in 2000, changed to a GVC in 2008 due to the entry of a public-agency-type shareholder—浙江新通留学有限公司 (Zhejiang Shinyway Education Group), and subsequently shifted to an IVC in 2014 because of the departure of the public-agency-type shareholder and the entry of two private firm shareholders. Nevertheless, due to the drastic increase in our sample size, collecting historical shareholder information of 49,187 VCs over 43 years would be financially costly. It might be economically more reasonable to remain cross-sectional also because the proportion of VCs that experienced type changes in VCAC is only 3.32% (i.e., 212 VCs). Among them, 174 VCs changed once, 31 changed twice, 4 changed three times, 2 changed four times, and 1 changed five times. In addition, only 72 had experienced a switch between a private type and a GVC type, accounting for 1.12% of VCAC’s whole sample. These suggest that in China, changes in VC types are uncommon. Generally, since VCs are light-asset companies and the fund management license is not scarce, registering a new VC is often more straightforward and less problematic than altering the type of an existing one. Consider the transition between GVCs and private VCs as an example. GVCs typically lack the incentive to become private because the primary objective of state capital in VCs is to attract private investment, not to convert private VCs into state-controlled VCs. Even if individuals within a GVC wish to adopt a private structure, the simplest way is to register a new, private VC firm, thereby avoiding potential issues related to state asset loss. Conversely, while some private fund management teams might acquire stakes in GVCs to improve fundraising from SOEs, this is an infrequent occurrence because it would be more flexible to form syndications with GVCs, thereby avoiding the complexities of ownership changes. Given this backdrop, it is logical for ChinaVCtype to remain a cross-sectional format, weighing the costs and benefits; nevertheless, this approach may not serve researchers interested in the dynamics of VC types.

To alleviate this concern, we offer insights into the dynamics of VC types using VCAC dataset, enabling researchers to utilize ChinaVCtype with caution. First, Table 13 reports the total times of changes for each type of VCs. It indicates that IVCs experienced the most changes compared to other types of VCs, followed by FVCs, GVCs, and CVCs. No PenVCs experienced a type change.

**Table 13:** The number of observations regarding changes in VC types in VCAC dataset.

	IVC	CVC	GVC	FVC	BVC	UVC	PenVC
Change from a focal type to another	103	46	53	57	1	1	0
Change from another type to a focal type	118	45	47	49	2	0	0
No change	101,859	101,989	101,980	101,974	102,077	102,079	102,080

The columns report the time of changes from a focal VC type to another, from another type to the focal type, and that remained unchanged, according to VCAC data. *Note*: A VC may change its type more than once.

Second, Table 14 provides more details regarding the direction of changes, indicating that the most frequent direction of changes is from FVC to IVC (45 times), followed by GVC to IVC (40 times) and IVC to FVC (40 times). Thus, studies on FVC, IVC, and GVCs should be more cautious about VC-type changes than studies on other types of VCs.

**Table 14:** The direction of changes in VC types in VCAC dataset.

	To IVC	To CVC	To GVC	To FVC	To BVC	To UVC	In total
From IVC	—	35	28	40	0	0	103
From CVC	33	—	11	2	0	0	46
From GVC	40	5	—	7	1	0	53
From FVC	45	5	6	—	1	0	57
From BVC	0	0	1	0	—	0	1
From UVC	0	0	1	0	0	—	1
In total	118	45	47	49	2	0	261

The columns report the direction of changes from one VC type to another, according to VCAC dataset. *Note*: A VC may change its type more than once.

Third, researchers may also be interested in the trend of changes. As illustrated in Table 15, although the instances of VCs changing their types seem to increase over time, peaking at 77 in 2016, the proportion of VCs undergoing type changes within the entire sample has remained very low. On the one hand, this finding alleviates the potential drawbacks of remaining ChinaVCtype cross-sectional; on the other hand, it does hint that studies on more recent VC markets shall be more careful in interpreting their results based on ChinaVCtype.

**Table 15:** The trend of changes in VC types in VCAC dataset.

Year	No. of changed VCs	Proportion of changed VCs in the whole sample
2000	0	0
2001	3	0.0005
2002	1	0.0002
2003	2	0.0003
2004	4	0.0006
2005	3	0.0005
2006	4	0.0006
2007	2	0.0003
2008	8	0.0013
2009	6	0.0009
2010	9	0.0014
2011	18	0.0028
2012	10	0.0016
2013	19	0.0030
2014	26	0.0041
2015	69	0.0108
2016	77	0.0121

The columns report the instances and proportion of VCs that experienced type changes, according to VCAC dataset.

## Data Availability

The program used for machine learning is Python. The codes can be accessed through GitHub (https://github.com/Emma-ning/ChinaVCtype).
